# The mRNA-1273 Vaccine Induces Cross-Variant Antibody Responses to SARS-CoV-2 With Distinct Profiles in Individuals With or Without Pre-Existing Immunity

**DOI:** 10.3389/fimmu.2021.737083

**Published:** 2021-09-03

**Authors:** Sonia Tejedor Vaquero, Leire de Campos-Mata, José María Ramada, Pilar Díaz, Juan Navarro-Barriuso, Clara Ribas-Llaurado, Natalia Rodrigo Melero, Carlo Carolis, Andrea Cerutti, Ramon Gimeno, Giuliana Magri

**Affiliations:** ^1^Translational Clinical Research Program, Institut Hospital del Mar d’Investigacions Mèdiques (IMIM), Barcelona, Spain; ^2^Epidemiology and Public Health, Institut Hospital del Mar d’Investigacions Mèdiques, Barcelona, Spain; ^3^CIBER de Epidemiología Y Salud Pública (CIBERESP), Barcelona, Spain; ^4^Center for Research in Occupational Health (CISAL), Institut Hospital del Mar d’Investigacions Mèdiques (IMIM), Barcelona, Spain; ^5^Centre for Genomic Regulation (CRG), The Barcelona Institute of Science and Technology, Barcelona, Spain; ^6^Pompeu Fabra University (UPF), Barcelona, Spain; ^7^Department of Medicine, Immunology Institute, Icahn School of Medicine at Mount Sinai, New York, NY, United States; ^8^Catalan Institute for Research and Advanced Studies (ICREA), Barcelona, Spain; ^9^Department of Pathology, Hospital del Mar, Barcelona, Spain; ^10^Department of Cell Biology, Physiology and Immunology, Universitat Autonoma de Barcelona, Barcelona, Spain

**Keywords:** humoral immunity, antibody subclasses, COVID-19, variants of concern, mRNA vaccination

## Abstract

mRNA-based vaccines effectively induce protective neutralizing antibodies against SARS-CoV-2, the etiological agent of COVID-19. Yet, the kinetics and compositional patterns of vaccine-induced antibody responses to the original strain and emerging variants of concern remain largely unknown. Here we characterized serum antibody classes and subclasses targeting the spike receptor-binding domain of SARS-CoV-2 wild type and α, β, γ and δ variants in a longitudinal cohort of SARS-CoV-2 naïve and COVID-19 recovered individuals receiving the mRNA-1273 vaccine. We found that mRNA-1273 vaccine recipients developed a SARS-CoV-2-specific antibody response with a subclass profile comparable to that induced by natural infection. Importantly, these antibody responses targeted both wild type SARS-CoV-2 as well as its α, β, γ and δ variants. Following primary vaccination, individuals with pre-existing immunity showed higher induction of all antibodies but IgG3 compared to SARS-CoV-2-naïve subjects. Unlike naïve individuals, COVID-19 recovered subjects did not mount a recall antibody response upon the second vaccine dose. In these individuals, secondary immunization resulted in a slight reduction of IgG1 against the receptor-binding domain of β and γ variants. Despite the lack of recall humoral response, vaccinees with pre-existing immunity still showed higher titers of IgG1 and IgA to all variants analyzed compared to fully vaccinated naïve individuals. Our findings indicate that mRNA-1273 vaccine triggered cross-variant antibody responses with distinct profiles in vaccinees with or without pre-existing immunity and suggest that individuals with prior history of SARS-CoV-2 infection may not benefit from the second mRNA vaccine dose with the current standard regimen.

## Introduction

Vaccination against Severe Acute Respiratory Syndrome Coronavirus 2 (SARS-CoV-2) is the leading strategy to achieve protection against coronavirus disease 19 (COVID-19) (1). SARS-CoV-2 messenger RNA (mRNA)-based vaccines, such as “mRNA-1273” from Moderna or “BNT162b2” from Pfizer-BioNTech are safe and highly effective in preventing severe COVID-19 in clinical trials (2, 3) and are currently being administered to millions of individuals worldwide. mRNA-1273 encodes for the full-length spike (S) protein of SARS-CoV-2, which is essential for viral pathogenicity, and is administered in a two-dose immunization regimen, with a 28-day inter-dose period.

Earlier phase III trials and more recent studies have demonstrated that mRNA-1273 vaccine induces a robust IgG response against SARS-CoV-2 wild type (WT) strain and promotes the generation of S-specific memory T and B cells after the two-dose regimen (2–8). Among the different antibodies induced by vaccination, those recognizing the S protein receptor-binding domain (RBD) are particularly relevant as they mirror the serum neutralizing capacity (9, 10), and are considered a main correlate of immune protection and vaccine efficacy (11).

Interestingly, SARS-CoV-2 naïve individuals and individuals with prior history of infection have distinct immune responses upon mRNA vaccination. Following the first vaccine dose, COVID-19 recovered individuals show significantly higher S- and RBD-specific IgG titers, superior serum neutralization activity (7, 12–14), and increased S protein-specific memory T and B cell responses than naïve individuals (4–6, 15). This enhanced response is consistent with the persistence of humoral and cellular immunity against SARS-CoV-2 in convalescent individuals, as previously reported (16–20).

These studies have highlighted the importance of understanding the immunological history of an individual to evaluate the efficacy of vaccines against SARS-CoV-2. However, to date, the analysis of humoral responses to SARS-CoV-2 vaccines has mostly focused on measuring S protein-specific total IgG responses. The contribution of virus-specific IgG subclasses and non-IgG antibody classes to vaccine-induced humoral responses remains elusive.

Recent studies have shown that IgG1 and IgG3 subclasses are strongly induced soon after SARS-CoV-2 infection, whereas IgG2 and IgG4 subclasses are induced to a much lesser extent (10, 16, 21). In recovered individuals, humoral and B cell memory responses appeared to be IgG1-dominated (16, 19). SARS-CoV-2 infection also induced virus-specific IgM, IgA1 and, to a lesser extent, IgA2, which all wane during convalescence (16).

Lately, several SARS-CoV-2 variants with increased transmissibility, such as the α, β, γ and δ variants of concern (VOCs), have emerged across the world (22–24). The α variant was first documented in the United Kingdom in September 2020, and soon spread to more than 50 countries. The β and γ variants were first reported in South Africa and Brazil, respectively. The δ variant, which was first identified in India, has overtaken the previously dominant α variant and will be the globally dominant strain over the coming months due to its increased transmissibility (25). These VOCs bear multiple mutations in the S protein, including the receptor-binding motif of the RBD, which may attenuate the efficacy of SARS-CoV-2 vaccines (26–29). Despite being decreased, neutralizing antibody responses against circulating VOCs have been reported following mRNA vaccination (30–32). Yet, the pattern and kinetics of vaccine-induced cross-reactive humoral responses recognizing emerging VOCs remain to be explored.

Here we characterized serum RBD-specific antibody classes and subclasses, including IgG1, IgG2, IgG3, IgG4, IgM, IgA, IgA1 and IgA2, in a longitudinal cohort of SARS-CoV-2 naïve and COVID-19 recovered individuals who received the mRNA-1273 vaccine. We found that mRNA-1273 triggered antibody responses to both WT SARS-CoV-2 as well as its α, β, γ and δ VOCs. We also detected striking differences in the profile and kinetics of RBD-specific antibody responses to WT SARS-CoV-2 and its VOCs according to prior history of infection.

## Material and Methods

### Ethics Statement

This study was approved by the Ethical Committee for Clinical Investigation of the Institut Hospital del Mar d’Investigacions Mèdiques (Number 2020/9621/I) in Barcelona, Spain. Written informed consent was obtained from all subjects prior to enrolment.

### Study Cohort

**Figure 1:**
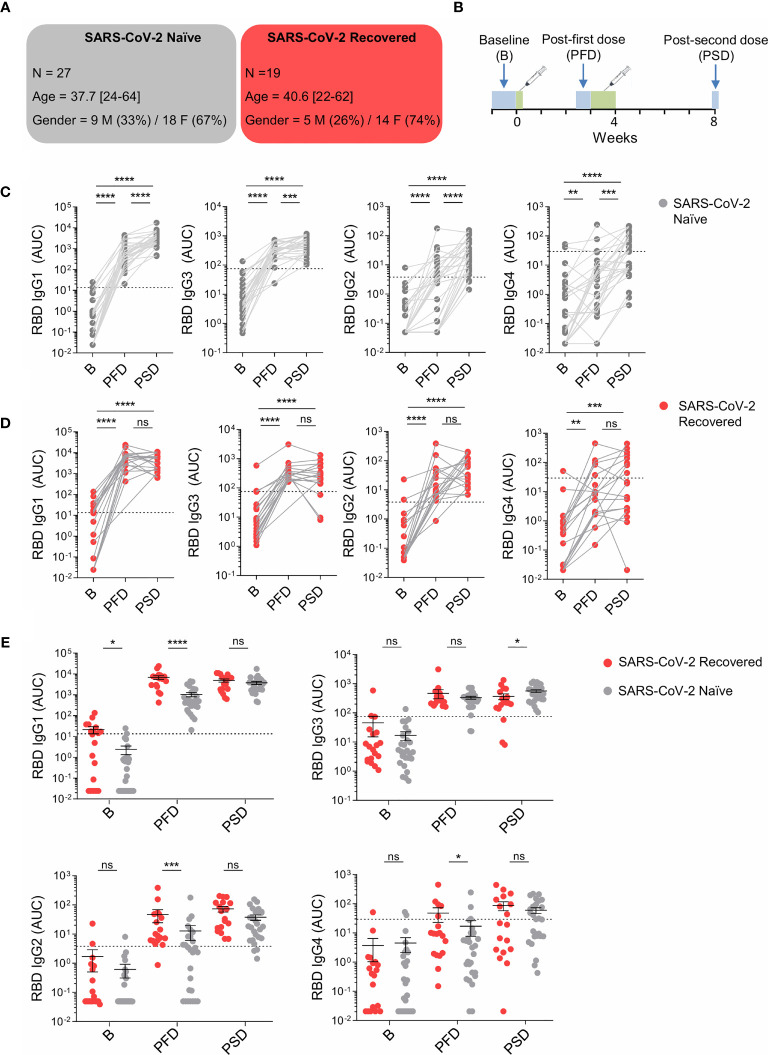
mRNA-1273 vaccination induces different pattern of WT RBD-specific IgG subclass responses in SARS-CoV-2 naïve and recovered individuals. **(A)** Schematic diagram of the cohort characteristics. M, male. F, female. **(B)** Schematic diagram of the study timeline. Sera were collected at 3 time points: pre-vaccine baseline, 2/3 weeks post the first dose (PFD) and 1 month post the second dose (PSD). Range of days per time point is indicated with a box. **(C)** Area under the curve (AUC) for each of the WT RBD-specific IgG subclasses analyzed from SARS-CoV-2 naïve individuals overtime. **(D)** AUC for each of the WT RBD-specific IgG subclasses analyzed from SARS-CoV-2 recovered individuals overtime. **(E)** AUC for each of the WT RBD-specific IgG subclasses analyzed from SARS-CoV-2 naïve and recovered individuals overtime depicted in the same graph. Sera from SARS-CoV-2 naïve individuals at baseline were used to establish negative threshold values defined as the naïve AUC mean plus 2 times the standard deviation of the mean. Bars represent mean ± standard error mean (SEM). Dashed line indicates negative threshold. Data are presented as individual dots. Wilcoxon matched pairs test was performed to compare time points. Two-tailed Mann-Whitney U test was performed to compare SARS-CoV-2 naïve and recovered individuals (ns, not significant P > 0.05, *P < 0.05, **P < 0.01, ***P < 0.001, and ****P < 0.0001). SARS-CoV-2 naïve, n=27; SARS CoV-2 recovered, n=19.

For this study, we recruited 46 healthy individuals among the health care personnel and research professionals of the Parc Salut Mar (Barcelona, Spain). Exclusion criteria included presence of symptoms suggestive of active SARS-CoV-2 infection and/or history of severe allergy. Subjects were stratified in two groups: SARS-CoV-2 naïve (n=27) and SARS-CoV-2 recovered (n=19), based on self-reported or laboratory evidence of prior SARS-CoV-2 infection. Of the self-reported naïve subjects, one individual was found to have positive SARS-CoV-2 nucleoprotein-specific antibodies at baseline and was retroactively classified as SARS-CoV-2 recovered. All subjects classified as SARS-CoV-2 recovered had mild COVID-19 course and symptoms during infection. Of those subjects, only one experienced fatigue and muscle aches for several months after infection. All participants provided survey data on prior SARS-CoV-2 infection, time elapsed from natural infection to mRNA vaccination and systemic side effects experienced after each vaccine dose. In the SARS-CoV-2 recovered group, 13 individuals had been infected more than six months before immunization, whereas 5 had been infected within the six months prior to vaccination. All study participants received the mRNA-1273 vaccine manufactured by Moderna, at Parc Salut Mar (Barcelona, Spain) between February and March 2021. Demographic information of participants and study design were summarized in **Figures 1A, B**. All collected sera were coded prior to processing and evaluation of antibody titers was performed in a blinded manner.

### Production of Recombinant SARS-CoV-2 Proteins

The pCAGGS RBD construct, encoding for the RBD of the WT SARS-CoV-2 S protein from the earliest lineage A virus (WT, YP_009724390.1, residues 319-541; NC_045512.2, A lineage), along with the signal peptide plus a hexahistidine tag was provided by Dr. Krammer (Mount Sinai School of Medicine, NY USA). RBD sequences from current α, β, and γ VOCs were obtained from the World Health Organization tracking of variants (https://www.who.int/en/activities/tracking-SARS-CoV-2-variants/) and Pango lineage classification (https://cov-lineages.org/). DNA fragments encoding the RBD from α (first identified in United Kingdom, B.1.1.7: N501Y), β (first identified in South Africa, B.1.351: K417N, E484K, N501Y) and γ (first identified in Japan/Brazil, P.1: K417T, E484K, N501Y) variants were synthetized by integrated DNA technology (IDT) as gblocks and codon optimized for mammalian expression. The fragments were inserted in a pCAGGS vector using Gibson Assembly. RBD proteins were expressed in-house in Expi293F human cells (Thermo Fisher Scientific) by transfection of the cells with purified DNA and polyethylenimine (PEI). RBD from δ variant (first identified in India, B.1.617.2: L452R, T478K) was purchased from Sino Biological.

### Enzyme-Linked Immunosorbent Assay

Sera were collected from whole blood, heat-inactivated at 56°C for 1 hour and stored at -20°C prior to use. ELISAs performed in this study were adapted from previously established protocols (21, 33). 96-well half-area flat bottom high-bind microplates (Corning) were coated overnight at 4°C with recombinant RBD from SARS-CoV-2 WT or VOCs (α, β γ and δ) at 2 µg/ml in PBS. Plates were washed with PBS 0.05% Tween 20 (PBS-T) and blocked with blocking buffer (PBS containing 1.5% bovine serum albumin, BSA) for 2 hours at room temperature (RT). Serum samples were serially diluted in PBS-T supplemented with 1% BSA and added to the viral protein-coated plates for 2 hours at RT. After washing, plates were incubated for 45 minutes at RT with horseradish peroxidase (HRP)-conjugated anti-human Ig secondary antibodies diluted in PBS-T supplemented with 1% BSA. To detect RBD-specific IgM and IgA, goat HRP-conjugated anti-human IgA and F(ab’)2 anti-human IgM (Southern Biotech) were used at a 1:4000 dilution. To measure RBD-specific IgG subclasses, HRP-conjugated mouse anti-human IgG1, IgG2, IgG3 and IgG4 (Southern Biotech) were used at a 1:3000 dilution. To detect RBD SARS-CoV-2-specific IgA1 and IgA2, HRP-conjugated mouse anti-human IgA1 or IgA2 (Southern Biotech) were used at a dilution of 1:2000 and 1:4000, respectively.

Plates were washed 5 times with PBS-T and developed by adding 50 µl of TMB substrate reagent set (BD bioscience). Then, the developing reaction was stopped with 50 µl 1M H_2_SO_4_. Absorbance was measured at 450 and 570 nm on a microplate reader (Infinite 200 PRO, Tecan). Optical density (OD) measurement was obtained after subtracting the absorbance at 570 nm from the absorbance at 450 nm. To quantitate the level of each viral antigen-specific antibody class or subclass, OD values from each dilution curves were used to determine the area under the curve (AUC). AUC values were computed by plotting OD values against the corresponding reciprocal serum sample dilutions using Prism 8 (GraphPad), as previously reported (33). Negative threshold values were set using healthy control AUC levels at baseline plus 2 times the standard deviations of the mean.

### Statistics

GraphPad Prism (version 8.0) was used to conduct statistical analyses. Data were analyzed for normal distribution (Shapiro-Wilk test) before any statistical analyses. Two-tailed unpaired Mann-Whitney U test was performed to compare SARS-CoV-2 naïve and recovered individuals at each specific time point. For comparison of two non-parametrically distributed paired datasets, we used the Wilcoxon matched-pairs signed rank test. Fisher’s exact test, two-sided, was performed to evaluate the association between previous history of infection and the appearance of side effects after primary vaccination. For each analysis, the type of statistical test, summary statistics and levels of significance were specified in the figures and corresponding legends. Missing data were not imputed. All tests were performed two-sided with a nominal significance threshold of p < 0.05.

## Results

For this study, we recruited 46 healthy individuals who received the mRNA-1273 SARS-CoV-2 vaccine from Moderna at Parc de Salut Mar in Barcelona, Spain. Of these individuals, 27 were SARS-CoV-2 naïve (mean age = 37.7; 67% female) and 19 had previous SARS-CoV-2 infection (mean age = 40.6; 74% female) (**Figure 1A**). Sera were collected at three time points: pre-vaccine baseline (B), 2/3 weeks post the first dose (PFD), and one month post the second dose (PSD; **Figure 1B**).

First, we evaluated the frequency of individuals who reported side effects such as fatigue, headache, chills, fever, adenopathy, intestinal symptoms or muscular pain following primary immunization, in SARS-CoV-2 naïve compared to recovered individuals. As previously shown (7, 14), a higher proportion of COVID-19 recovered subjects reported side effects after the first dose of Moderna mRNA vaccine compared to naïve individuals (**Supplementary Figure 1A**).

### IgG Subclass Response to WT SARS-CoV-2 in mRNA-1273 Vaccine Recipients

We measured the induction of WT RBD-specific IgG subclasses upon mRNA-1273 vaccination in SARS-CoV-2 naïve individuals and individuals with prior history of SARS-CoV-2 infection. The administration of the first vaccine dose in naïve individuals induced the production of all RBD-specific IgG subclasses, which further increased following the second dose. The highest increase was observed for RBD-specific IgG1 and, to a lesser extent, RBD-specific IgG3, whereas RBD-specific IgG2 and IgG4 showed much lower increases (**Figure 1C**). Interestingly, we did not observe increased titers of RBD-specific IgG1 following primary immunization in SARS-CoV-2 naïve vaccinees experiencing side effects compared to the ones that had no systemic reactions (**Supplementary Figure 1B**).

In subjects with pre-existing immunity, we also observed a significant increase of all RBD-specific IgG subclasses after the first dose of the mRNA vaccine; however, in these individuals, the booster dose did not further increase RBD-specific IgG subclasses (**Figure 1D**).

We then compared RBD-specific IgG1, IgG3, IgG2 and IgG4 present at baseline and induced 2/3 weeks following the first dose or one month following the second dose in SARS-CoV-2 naïve and recovered individuals (**Figure 1E**). At baseline, study participants with prior history of infection showed significantly higher levels of RBD-specific IgG1 compared to SARS-CoV-2 naïve individuals. In contrast, no significant differences were observed between these two groups with respect to other IgG subclasses. After the first dose of mRNA-1273 vaccine, RBD-specific IgG1, IgG2 and IgG4 titers were significantly higher in recovered individuals than in vaccinees with no pre-existing immunity to SARS-CoV-2. Interestingly, after primary immunization, the amount of RBD-specific IgG3 was similar in SARS-CoV-2 naïve and recovered individuals. Following the second dose of the mRNA vaccine, the concentrations of WT RBD-specific IgG1, IgG2, IgG4 were comparable between the two groups of vaccinees, whereas RBD-specific IgG3 were slightly higher in individuals with no prior history of SARS-CoV-2 infection compared to recovered individuals (**Figure 1E**). Of note, we did not observe any relationship between gender and RBD-specific IgG subclasses at baseline, after the first immunization and after the recall immunization (**Supplementary Figure 2A**), which is consistent with published findings (32, 34).

### IgM and IgA Subclass Responses to WT SARS-CoV-2 in mRNA Vaccine Recipients

**Figure 2:**
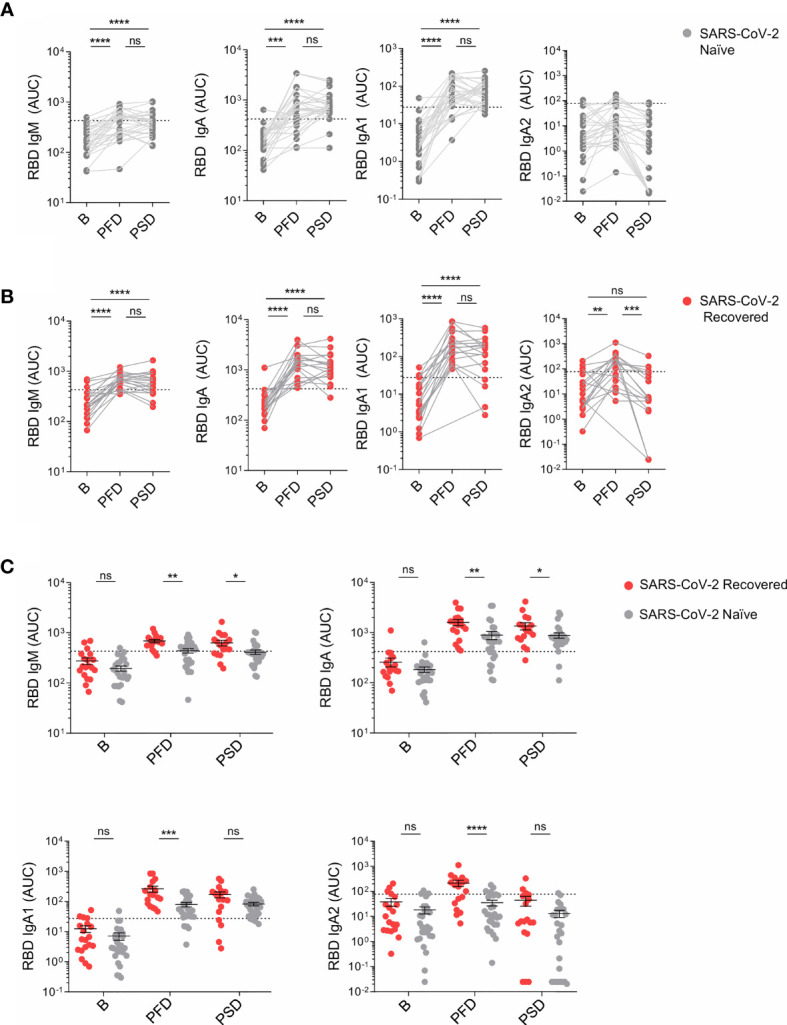
mRNA-1273 primary immunization triggers increased levels of WT RBD-specific IgM, IgA and IgA subclasses in recovered individuals compared to SARS-CoV-2 naïve individuals. **(A)** Area under the curve (AUC) for each of the WT RBD-specific IgM, IgA and IgA subclasses analyzed from SARS-CoV-2 naïve individuals overtime. **(B)** AUC for each of the WT RBD-specific IgM, IgA, and IgA subclasses analyzed from SARS-CoV-2 recovered individuals overtime. **(C)** AUC for each of the WT RBD-specific IgM, IgA and IgA subclasses analyzed from SARS-CoV-2 naïve and recovered individuals overtime depicted in the same graph. Sera from SARS-CoV-2 naïve individuals at baseline were used to establish negative threshold values defined as the naïve AUC mean plus 2 times the standard deviation of the mean. Bars represent mean ± standard error mean (SEM). Dashed line indicates negative threshold. Data are presented as individual dots. Wilcoxon matched pairs test was performed to compare time points. Two-tailed Mann-Whitney U test was performed to compare SARS-CoV-2 naïve and recovered individuals (ns, not significant P > 0.05, *P < 0.05, **P < 0.01, ***P < 0.001, and **** P< 0.0001). SARS-CoV-2 naïve, n=27; SARS CoV-2 recovered, n=19.

We further analyzed whether S-specific IgM as well as IgA, IgA1 and IgA2 subclasses were differentially induced upon vaccination in SARS-CoV-2 naïve and COVID-19 recovered individuals. Our analysis revealed that RBD-specific IgM and IgA titers increased after primary vaccination, but they did not further augment upon reboost in both groups of vaccinees (**Figures 2A, B**). Similar to RBD-specific IgM, RBD-specific IgA1 was induced upon the first vaccine dose in SARS-CoV-2 naïve and recovered individuals, but its serum concentration no longer augmented PSD (**Figures 2A, B**). In SARS-CoV-2 naïve individuals, RBD-specific IgA2 titers were below the threshold in all time points studied (**Figure 2A**), whereas a small but significant induction was observed in individuals with pre-existing immunity after primary but not secondary vaccination (**Figure 2B**). When we compared the levels of RBD-specific IgM, IgA1 and IgA2 in SARS-CoV-2 naïve and recovered individuals, we observed no significant differences at baseline, being most of AUC values below the threshold. However, following the first and second vaccine dose, individuals with prior history of SARS-CoV-2 infection showed significantly higher levels of RBD-specific IgM and IgA compared to SARS-CoV-2 naïve individuals (**Figure 2C**). No association was observed between gender and RBD-specific IgM or IgA subclasses following primary and secondary immunization (**Supplementary Figure 2B**).

### Antibody Responses to SARS-CoV-2 VOCs in mRNA-1273 Vaccine Recipients

**Figure 3:**
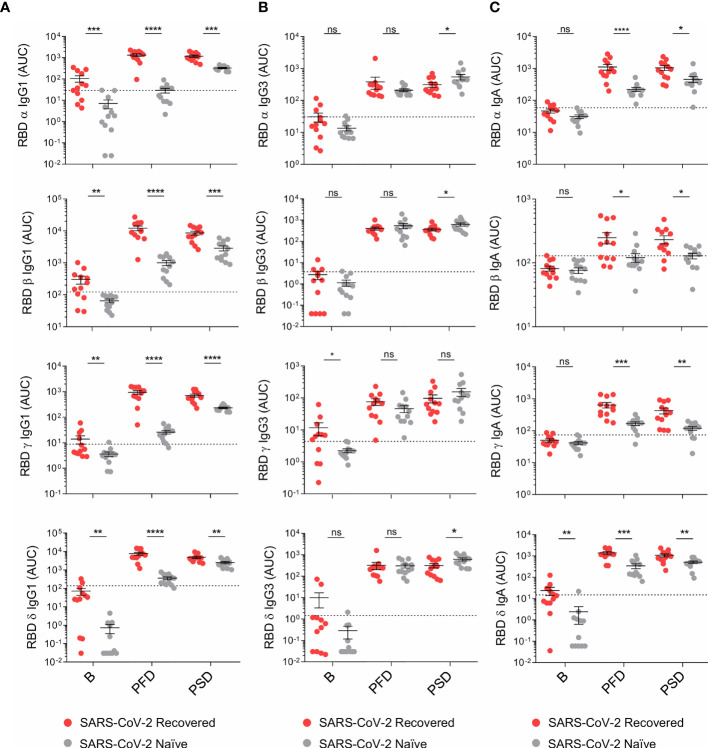
mRNA-1273 vaccination induces increased levels of cross-reactive IgG1 and IgA against the variants of concern in recovered individuals compared to SARS-CoV-2 naïve individuals. **(A)** AUC for the IgG1, **(B)** IgG3, and **(C)** IgA antibodies against the RBD of the α, β, γ and δ variants analyzed from SARS-CoV-2 naïve and recovered individuals overtime. Sera from SARS-CoV-2 naïve individuals at baseline were used to establish negative threshold values defined as the naïve AUC mean plus 2 times the standard deviation of the mean. Bars represent mean ± standard error mean (SEM). Dashed line indicates negative threshold. Data are presented as individual dots. Two-tailed Mann-Whitney U test was performed to compare SARS-CoV-2 naïve and recovered individuals (ns, not significant P > 0.05, *P < 0.05, **P < 0.01, ***P < 0.001, and ****P < 0.0001). SARS-CoV-2 naïve, n=12; SARS CoV-2 recovered, n=12.

Lastly, we analyzed vaccine-induced IgG1, IgG3 and IgA to the RBD of α, β, γ and δ VOCs (**Figure 3**). In individuals with prior history of infection, primary vaccination increased the titers of cross-reactive IgG1, IgG3 and IgA to the RBD of α, β, γ and δ VOCs. However, these responses were not boosted by the second vaccine dose (**Supplementary Figure 3**). Moreover, IgG1 targeting the RBD of β and γ variants slightly decreased following the second dose (**Supplementary Figure 3A**). mRNA-1273 immunization of naïve individuals also induced cross-reactive antibodies to both WT SARS-CoV-2 and VOCs which further augmented following the second vaccine dose. Despite this reboost, fully vaccinated naïve individuals still showed lower titers of cross-reactive IgG1 and IgA binding the RBD of VOCs compared to recovered individuals (**Figures 3A, C**). Conversely, after the two-dose regimen, the titers of IgG3 to VOCs were either comparable or slightly higher in SARS-CoV-2 naïve individuals compared to vaccinees with prior history of infection (**Figure 3B**).

## Discussion

Here, we analyzed the pattern and kinetics of the antibody responses induced by the mRNA-1273 vaccine in a cohort of healthy SARS-CoV-2 naïve and COVID-19 recovered individuals. Our findings highlight both the complexity and heterogeneity of antibody responses against SARS-CoV-2 WT and α, β, γ and δ VOCs following mRNA vaccination in individuals with different exposure history.

Following primary immunization, individuals with pre-existing immunity showed increased titers of all antibodies except IgG3 against WT and VOCs, when compared to SARS-CoV-2 naïve subjects. Our data were consistent with recent studies that reported the presence of pre-existing SARS-CoV-2-specific and cross-variant specific-memory B cells in COVID-19 recovered subjects (16, 17, 19, 20, 35). In line with our results, a recent report showed a strong correlation between the frequency of SARS-CoV-2-specific memory B cells at baseline and the antibody concentrations following vaccination (6). Moreover, the lack of superior RBD-specific IgG3 responses in COVID-19 recovered individuals is in agreement with earlier findings that reported a low frequency of SARS-CoV-2 memory B cells expressing IgG3 in COVID-19 convalescent individuals (19). Interestingly, we also found that the lack of systemic side effects after the first vaccine dose did not associate with diminished RBD-specific IgG1 responses in SARS-CoV-2 naïve individuals, suggesting that vaccine-induced humoral immunity is indeed independent of reactogenicity.

Furthermore, we observed that primary vaccination induced a broad spectrum of virus-reactive antibody responses, which included RBD-specific IgM, IgA, IgA1 as well as IgG1, IgG3 and, to a lesser extent, IgG2 and IgG4. Keeping in mind the largely mucosal nature of IgA2 (36), it was not surprising to detect virtually no RBD-specific IgA2 induction in individuals with no prior history of infection. Thus, except for the lack of IgA2, the general pattern of subclass-specific antibody responses induced by primary immunization with mRNA-1273 vaccine in SARS-CoV-2 naïve individuals was comparable to that seen upon natural infection (14). The small but significant induction of RBD-specific IgA2 in recovered individuals upon the first dose of mRNA vaccine echoes earlier studies that describe IgA2-expressing memory B cells targeting the S protein of SARS-CoV-2 in a subset of COVID-19 recovered patients (37, 38).

Of note, differences in vaccine-induced antibody responses among SARS-CoV-2 naïve subjects and COVID-19 recovered individuals were also present following secondary immunization. Unlike individuals with prior history of infection, naïve subjects showed a vigorous boosting of RBD-specific IgG responses upon the second dose of the mRNA vaccine, which involved all IgG subclasses. Moreover, in naïve individuals, the recall immunization increased cross-reactive IgG1 against the SARS-CoV-2 VOCs analyzed. Yet, fully vaccinated naïve individuals still showed significantly lower titers of IgG1 and IgA to the RBD of VOCs, whereas IgG3 binding the RBD of WT SARS-CoV-2 or α and β VOCs were significantly higher. Considering the increased VOC-specific IgG1 in recovered individuals and the putative lack of IgG3 long-term memory (19), fully vaccinated SARS-CoV-2 naïve individuals might remain more vulnerable to infection by those VOCs than vaccinees with prior SARS-CoV-2 exposure history. Consistent with this hypothesis, a recent study showed that mRNA vaccination can boost cross-variant neutralizing antibodies more efficiently in previously infected individuals than in SARS-CoV-2 naïve individuals (30).

The lack of increased antibody responses following recall vaccination in individuals with pre-existing immunity to SARS-CoV-2 is intriguing and could relate to the reduced antigen availability resulting from the elevated vaccine-specific IgG responses triggered by the primary immunization. However, it could also reflect differences in the kinetics of germinal center or extra follicular B cell responses to viral antigens as well as differences in priming of antigen-presenting cells. Such differences may influence the generation of secretory memory, including the generation of long-lived plasma cells in the bone marrow.

Interestingly, secondary immunization of individuals with prior history of infection resulted in a slight reduction of RBD-specific IgG1 to β and γ VOCs, which might be associated with diminished serum neutralizing activity against these VOCs. This result raises the possibility that secondary mRNA-1273 immunization of individuals with prior history of infection leads to the positive selection of IgG1-expressing memory and plasma cells with higher affinity for the WT form of the S protein. This putative effect could attenuate cross-variant neutralizing antibodies elicited by natural infection. Further long-term studies are required to address these questions.

In summary, our findings show that mRNA-1273 elicits humoral responses against WT SARS-CoV-2 as well as α, β, γ and δ VOCs with antibody class and subclass profiles that are comparable to those induced by natural infection. Yet, the pattern and kinetics of vaccine-induced antibody responses differed between individuals with or without pre-existing immunity. In addition, the present data suggest that individuals with prior SARS-CoV-2 infection may not benefit from the second mRNA-1273 vaccine dose, as per the current standard regimen, which may prompt to re-think both vaccination schedule and dose distribution.

### Limitations of the Study

This study has the following limitations. First, the size of the cohort was small (*n* = 46) and all the participants were below 65 years of age (mean = 39). Hence, our results should be confirmed in future larger-scale studies including older individuals to fully explore vaccine-induced humoral responses. Moreover, to further investigate the protection offered by mRNA vaccines, virus-specific B cell memory with subclasses resolution should be assessed. Finally, additional long-term studies that integrate the analysis of humoral responses with the neutralization activity and Fc-mediated function of vaccine-induced antibodies may be performed to define new correlates of protection against symptomatic SARS-CoV-2 infection.

## Data Availability Statement

The raw data supporting the conclusions of this article will be made available by the authors, without undue reservation.

## Ethics Statement

The studies involving human participants were reviewed and approved by the Ethical Committee for Clinical Investigation of the Institut Hospital del Mar d’Investigacions Mèdiques (Number 2020/9621/I). The patients/participants provided their written informed consent to participate in this study.

## Author Contributions

STV and LDC-M performed experiments, analyzed and discussed data and wrote the manuscript. JMR, PD, JN-B and CR-L recruited study participants and processed samples. NRM and CC produced recombinant SARS-CoV-2 antigens. AC participated in data analysis and interpretation and wrote the manuscript. RG conceived the study, recruited study participants, discussed data and wrote the manuscript. GM conceived the study and designed experiments, analyzed results, discussed data, and wrote the manuscript. All authors contributed to the article and approved the submitted version.

## Funding

This study was supported by the COVID-19 call grant from Generalitat de Catalunya, Department of Health (to GM), grant Miguel Servet research program (to GM) and by National Health Institute Carlos III (ISCIII) through the project COV20_00508 grant (Co-funded by European Regional Development Fund/European Social Fund “a way to make Europe) (to RG).

## Conflict of Interest

The authors declare that the research was conducted in the absence of any commercial or financial relationships that could be construed as a potential conflict of interest.

## Publisher’s Note

All claims expressed in this article are solely those of the authors and do not necessarily represent those of their affiliated organizations, or those of the publisher, the editors and the reviewers. Any product that may be evaluated in this article, or claim that may be made by its manufacturer, is not guaranteed or endorsed by the publisher.
