# Supplementary material for: The mRNA-1273 Vaccine Induces Cross-Variant Antibody Responses to SARS-CoV-2 With Distinct Profiles in Individuals With or Without Pre-Existing Immunity
Source: Front Immunol. 2021 Sep 3;12:737083. doi: 10.3389/fimmu.2021.737083 (PMC8446508; doi:10.3389/fimmu.2021.737083)
Supplement: Supplementary file 1 [file DataSheet_1.pdf]

Supplementary Material

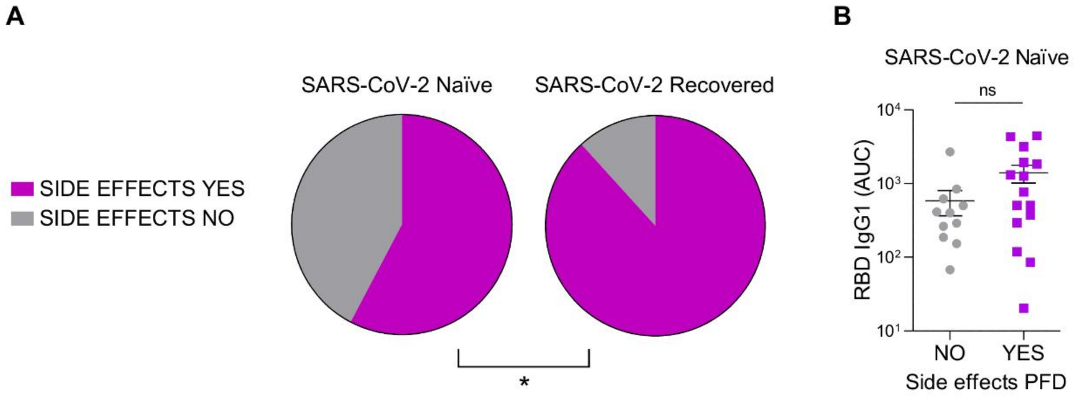

Supplementary Figure 1. SARS-CoV-2 recovered individuals experience side effects in higher proportion that naïve individuals upon primary immunization. (**A**) Distribution of SARS-CoV-2 naïve (left) and recovered (right) individuals that experienced side effects upon primary immunization. SARS-CoV-2 naïve individuals: side effects yes (*n*=15), side effects no (*n*=11). SARS-CoV-2 recovered individuals: side effects yes (n=15), side effects no (n=2). Fisher's exact test, two-sided, was performed to compare the distribution of SARS-CoV-2 naïve and recovered individuals with side effects. (**B**) AUC for the RBD-specific IgG1 subclass analyzed from SARS-CoV-2 naïve individuals segregated according to the presence (YES) or absence (NO) of side effects upon primary immunization. Bars represent mean ± standard error mean (SEM). Two-tailed Mann-Whitney U test was performed to compare antibody responses in naïve individuals with and without side effects (ns, non-significant ($P > 0.05$), *$P < 0.05$, **$P < 0.01$, ***$P < 0.001$, and ****$P < 0.0001$). SARS-CoV-2 naïve, n=26; SARS CoV-2 recovered, n=17.

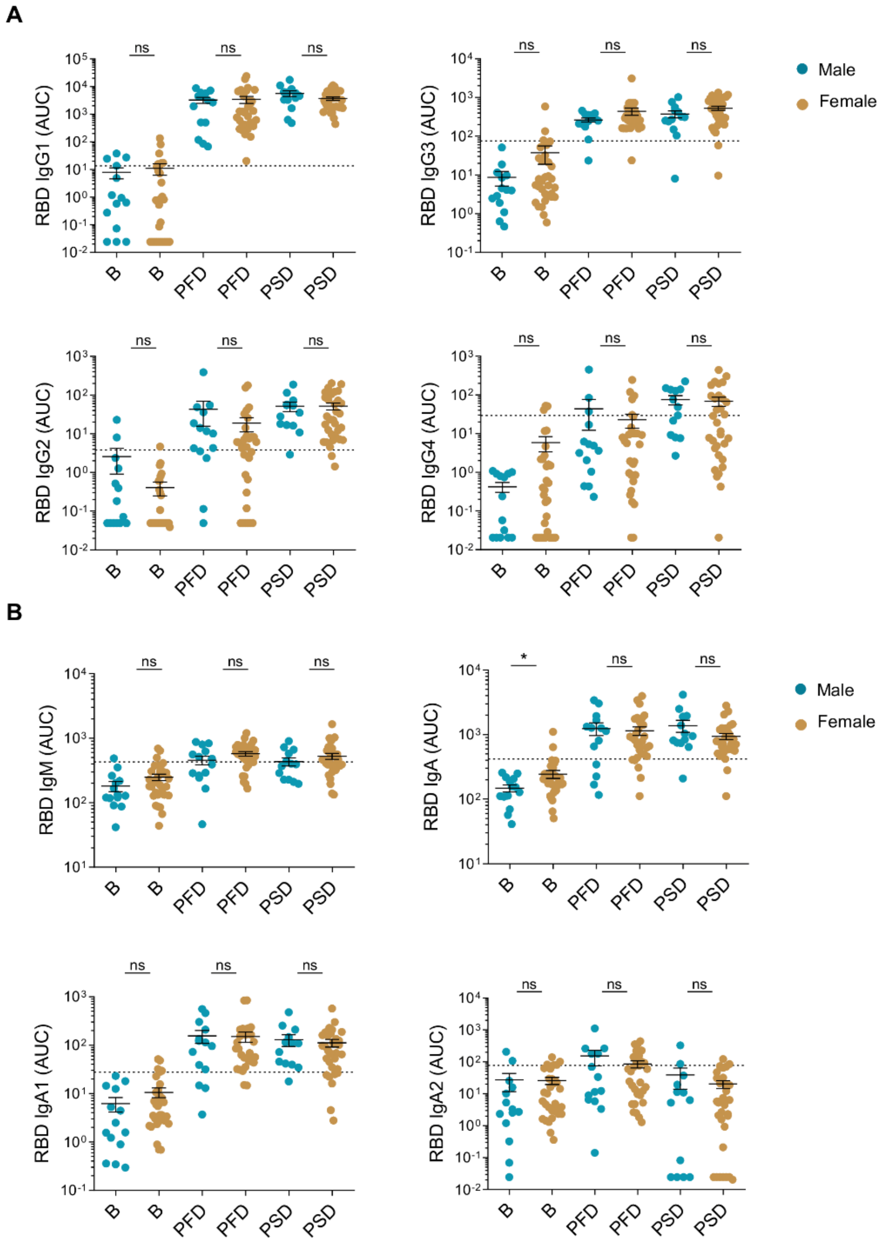

Supplementary Figure 2. mRNA-1273 vaccination induces antibody responses irrespective of the gender of the individuals. (**A**) Area under the curve (AUC) for each of the WT RBD-specific IgG subclasses analyzed from male and female individuals overtime. (**B**) Area under the curve (AUC) for each of the WT RBD-specific IgM, IgA and IgA subclasses analyzed from male and female individuals overtime. SARS-CoV-2 naïve individuals at baseline were used to establish negative threshold values defined as the naïve AUC mean plus 2 times the standard deviation of the mean. Bars represent mean ± standard error mean (SEM). Dashed line indicates negative threshold. Data are presented as individual dots. Two-tailed Mann-Whitney U test was performed to compare antibody responses between male and female (ns, non-significant ($P > 0.05$), *$P < 0.05$, **$P < 0.01$, and ***$P < 0.001$). Male, n=14; Female, n=32.

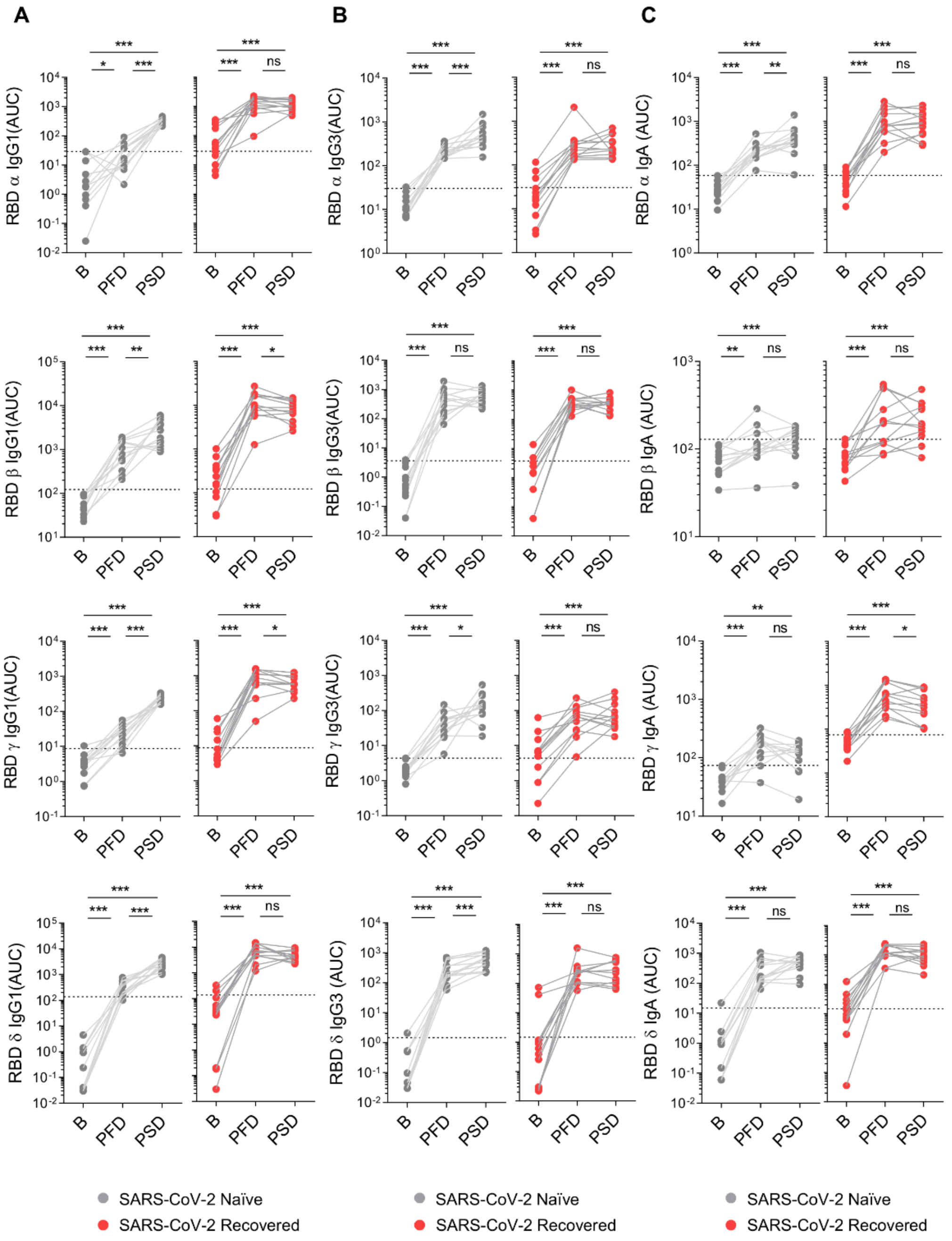

Supplementary Figure 3. mRNA-1273 vaccination does not trigger a recall response in SARS-CoV-2 recovered individuals against the variants of concern. (**A**) AUC for IgG1, (**B**) IgG3, and (**C**) IgA antibodies against the RBD of α, β, γ and δ variants analyzed from SARS-CoV-2 naïve (left) and recovered (right) individuals overtime. Sera from SARS-CoV-2 naïve individuals at baseline were used to establish negative threshold values defined as the naïve AUC mean plus 2 times the standard deviation of the mean. Dashed line indicates negative threshold. Data are presented as individual dots. Wilcoxon matched pairs test was performed to compare time points (ns, non-significant ($P > 0.05$), *$P < 0.05$, **$P < 0.01$, ***$P < 0.001$, and ****$P < 0.0001$). SARS-CoV-2 naïve, n=12; SARS CoV-2 recovered, n=12.
